# Supplementary material for: Unveiling the Bioactive Potential of the Invasive Jellyfish Phyllorhiza punctata Through Integrative Transcriptomic and Proteomic Analyses
Source: Biomolecules. 2025 Aug 4;15(8):1121. doi: 10.3390/biom15081121 (PMC12383608; doi:10.3390/biom15081121)
Supplement: Supplementary file 1 [file biomolecules-15-01121-s001.zip › Suplementary Material/FileS3/PEPFOLD4/XP_065063865_6239/clusters.html]

 PEP-FOLD clusters


Download archive of all models  here   

|  |  |  |
| --- | --- | --- |
| # | file | sOPEP |
| # cluster 1 - Size: 15 |  |  |
|  | XP\_065063865\_6239-model1 | -8.62671 |
|  | XP\_065063865\_6239-model1.1 | -8.62671 |
|  | XP\_065063865\_6239-model1.2 | -8.42122 |
|  | XP\_065063865\_6239-model1.3 | -8.42122 |
|  | XP\_065063865\_6239-model1.4 | -8.42122 |
|  | XP\_065063865\_6239-model1.5 | -8.31338 |
|  | XP\_065063865\_6239-model1.6 | -8.31338 |
|  | XP\_065063865\_6239-model1.7 | -8.25951 |
|  | XP\_065063865\_6239-model1.8 | -8.2287 |
|  | XP\_065063865\_6239-model1.9 | -8.2287 |
|  | XP\_065063865\_6239-model1.10 | -8.20748 |
|  | XP\_065063865\_6239-model1.11 | -8.18383 |
|  | XP\_065063865\_6239-model1.12 | -8.13953 |
|  | XP\_065063865\_6239-model1.13 | -8.0632 |
|  | XP\_065063865\_6239-model1.14 | -8.02833 |
| # cluster 2 - Size: 10 |  |  |
|  | XP\_065063865\_6239-model2 | -8.44418 |
|  | XP\_065063865\_6239-model2.1 | -8.44418 |
|  | XP\_065063865\_6239-model2.2 | -8.44418 |
|  | XP\_065063865\_6239-model2.3 | -8.44418 |
|  | XP\_065063865\_6239-model2.4 | -8.44418 |
|  | XP\_065063865\_6239-model2.5 | -8.44418 |
|  | XP\_065063865\_6239-model2.6 | -8.44418 |
|  | XP\_065063865\_6239-model2.7 | -8.23838 |
|  | XP\_065063865\_6239-model2.8 | -8.20447 |
|  | XP\_065063865\_6239-model2.9 | -7.75982 |
| # cluster 3 - Size: 5 |  |  |
|  | XP\_065063865\_6239-model3 | -8.36538 |
|  | XP\_065063865\_6239-model3.1 | -8.36538 |
|  | XP\_065063865\_6239-model3.2 | -8.33549 |
|  | XP\_065063865\_6239-model3.3 | -8.33549 |
|  | XP\_065063865\_6239-model3.4 | -8.32461 |
| # cluster 4 - Size: 66 |  |  |
|  | XP\_065063865\_6239-model4 | -8.35614 |
|  | XP\_065063865\_6239-model4.1 | -8.35614 |
|  | XP\_065063865\_6239-model4.2 | -8.35614 |
|  | XP\_065063865\_6239-model4.3 | -8.35614 |
|  | XP\_065063865\_6239-model4.4 | -8.35614 |
|  | XP\_065063865\_6239-model4.5 | -8.35614 |
|  | XP\_065063865\_6239-model4.6 | -8.35614 |
|  | XP\_065063865\_6239-model4.7 | -8.35614 |
|  | XP\_065063865\_6239-model4.8 | -8.35614 |
|  | XP\_065063865\_6239-model4.9 | -8.35614 |
|  | XP\_065063865\_6239-model4.10 | -8.35614 |
|  | XP\_065063865\_6239-model4.11 | -8.35614 |
|  | XP\_065063865\_6239-model4.12 | -8.35614 |
|  | XP\_065063865\_6239-model4.13 | -8.35614 |
|  | XP\_065063865\_6239-model4.14 | -8.35614 |
|  | XP\_065063865\_6239-model4.15 | -8.35614 |
|  | XP\_065063865\_6239-model4.16 | -8.3494 |
|  | XP\_065063865\_6239-model4.17 | -8.30967 |
|  | XP\_065063865\_6239-model4.18 | -8.30967 |
|  | XP\_065063865\_6239-model4.19 | -8.30967 |
|  | XP\_065063865\_6239-model4.20 | -8.30967 |
|  | XP\_065063865\_6239-model4.21 | -8.30967 |
|  | XP\_065063865\_6239-model4.22 | -8.30967 |
|  | XP\_065063865\_6239-model4.23 | -8.30967 |
|  | XP\_065063865\_6239-model4.24 | -8.30967 |
|  | XP\_065063865\_6239-model4.25 | -8.30967 |
|  | XP\_065063865\_6239-model4.26 | -8.27812 |
|  | XP\_065063865\_6239-model4.27 | -8.27812 |
|  | XP\_065063865\_6239-model4.28 | -8.27812 |
|  | XP\_065063865\_6239-model4.29 | -8.27812 |
|  | XP\_065063865\_6239-model4.30 | -8.27812 |
|  | XP\_065063865\_6239-model4.31 | -8.27812 |
|  | XP\_065063865\_6239-model4.32 | -8.27812 |
|  | XP\_065063865\_6239-model4.33 | -8.27812 |
|  | XP\_065063865\_6239-model4.34 | -8.27812 |
|  | XP\_065063865\_6239-model4.35 | -8.27812 |
|  | XP\_065063865\_6239-model4.36 | -8.27812 |
|  | XP\_065063865\_6239-model4.37 | -8.27812 |
|  | XP\_065063865\_6239-model4.38 | -8.25839 |
|  | XP\_065063865\_6239-model4.39 | -8.25839 |
|  | XP\_065063865\_6239-model4.40 | -8.22257 |
|  | XP\_065063865\_6239-model4.41 | -8.22257 |
|  | XP\_065063865\_6239-model4.42 | -8.22257 |
|  | XP\_065063865\_6239-model4.43 | -8.22257 |
|  | XP\_065063865\_6239-model4.44 | -8.22257 |
|  | XP\_065063865\_6239-model4.45 | -8.21788 |
|  | XP\_065063865\_6239-model4.46 | -8.21788 |
|  | XP\_065063865\_6239-model4.47 | -8.18918 |
|  | XP\_065063865\_6239-model4.48 | -8.18918 |
|  | XP\_065063865\_6239-model4.49 | -8.18918 |
|  | XP\_065063865\_6239-model4.50 | -8.18918 |
|  | XP\_065063865\_6239-model4.51 | -8.17333 |
|  | XP\_065063865\_6239-model4.52 | -8.17333 |
|  | XP\_065063865\_6239-model4.53 | -8.17333 |
|  | XP\_065063865\_6239-model4.54 | -8.17333 |
|  | XP\_065063865\_6239-model4.55 | -8.17333 |
|  | XP\_065063865\_6239-model4.56 | -8.16634 |
|  | XP\_065063865\_6239-model4.57 | -8.12382 |
|  | XP\_065063865\_6239-model4.58 | -8.12382 |
|  | XP\_065063865\_6239-model4.59 | -8.11656 |
|  | XP\_065063865\_6239-model4.60 | -8.0571 |
|  | XP\_065063865\_6239-model4.61 | -8.04849 |
|  | XP\_065063865\_6239-model4.62 | -8.04849 |
|  | XP\_065063865\_6239-model4.63 | -8.04179 |
|  | XP\_065063865\_6239-model4.64 | -7.88242 |
|  | XP\_065063865\_6239-model4.65 | -7.70836 |
| # cluster 5 - Size: 2 |  |  |
|  | XP\_065063865\_6239-model5 | -7.92981 |
|  | XP\_065063865\_6239-model5.1 | -7.7041 |
| # cluster 6 - Size: 2 |  |  |
|  | XP\_065063865\_6239-model6 | -7.40755 |
|  | XP\_065063865\_6239-model6.1 | -7.11551 |
|  |  |  |
